# Comprehensive evaluation of blood-brain barrier-forming micro-vasculatures: Reference and marker genes with cellular composition

**DOI:** 10.1371/journal.pone.0197379

**Published:** 2018-05-15

**Authors:** Mei Dai, Yi Lin, Salim S. El-Amouri, Mara Kohls, Dao Pan

**Affiliations:** 1 Division of Experimental Hematology & Cancer Biology, Cincinnati Children’s Hospital Medical Center, Cincinnati, Ohio, United States of America; 2 Department of Pediatrics, School of Medicine, University of Cincinnati, Cincinnati, Ohio, United States of America; Hungarian Academy of Sciences, HUNGARY

## Abstract

Primary brain microvessels (BrMV) maintain the cellular characters and molecular signatures as displayed in vivo, and serve as a vital tool for biomedical research of the blood-brain barrier (BBB) and the development/optimization of brain drug delivery. The variations of relative purities or cellular composition among different BrMV samples may have significant consequences in data interpretation and research outcome, especially for experiments with high-throughput genomics and proteomics technologies. In this study, we aimed to identify suitable reference gene (RG) for accurate normalization of real-time RT-qPCR analysis, and determine the proper marker genes (MG) for relative purity assessment in BrMV samples. Out of five housekeeping genes, β-actin was selected as the most suitable RG that was validated by quantifying mRNA levels of alpha-L-iduronidase in BrMV isolated from mice with one or two expressing alleles. Four marker genes highly/selectively expressed in BBB-forming capillary endothelial cells were evaluated by RT-qPCR for purity assessment, resulting in *Cldn*5 and *Pecam1* as most suitable MGs that were further confirmed by immunofluorescent analysis of cellular components. *Plvap* proved to be an indicator gene for the presence of fenestrated vessels in BrMV samples. This study may contribute to the building blocks toward overarching research needs on the blood-brain barrier.

## Introduction

The blood-brain barrier (BBB) plays a key role in normal neuronal function, disease and aging as it serves the needs for homeostasis, entry of nutrients, and communication of the central nervous system (CNS)[1, 2]. BBB remodeling has been indicated in the pathogenesis of various CNS diseases such as neuronopathic lysosomal storage diseases or Alzheimer's disease[3, 4]. Moreover, the neuroprotective role of the BBB hinders rapid and wide systemic delivery of therapeutic drugs or diagnostic agents to the CNS[5]. The BBB comprises approximately 600 km of capillaries formed by highly specialized brain capillary endothelial cells (BrEC), which are characterized by less pinocytotic activity, a lack of fenestrations, and unique expression patterns of trans-membrane transport receptors[6]. Inter-endothelial tight junctions form a physical barrier that blocks the contents of blood from entering the brain through para-cellular routes [7]. Astrocytic end-feet surround more than 90% of the BrEC abluminal surface and, together with pericytes, microglial cells, and neuronal endings, form the neurovascular unit and influence the “tightness” and trafficking function of the barrier[8]. The complexity of BBB architecture heightens the difficulty of developing BBB models that capture physical and biological characteristics of the BBB in a physiologically relevant geometry, thus contributing to the paucity of therapies and diagnoses for most neurological disorders[3]. Inconclusive or sometimes contradictory results in BBB-related studies are attributable, in part, to a shortage of standardized and systematically validated evaluation tools.

Two widely used approaches for studying the BBB in vitro include brain endothelial cell culture-based models and the utilization of isolated primary brain microvessels (BrMV). While an ultimate cell culture system that possesses all major BrEC properties remains to be developed [9, 10], microvessels freshly isolated from the brain largely retain their functional and cellular characters and molecular signatures as displayed *in vivo*, which is critical for the BBB research and the development/optimization of brain drug delivery[11]. However, due to the unique structure of the BBB, contamination of BrMV with cells other than BrEC is unavoidable, regardless of isolation methods involving either mechanical[12], enzymatic[13] or laser microdissection[14] techniques. The variations in relative purity or cellular composition among different BrMV isolates may have significant consequences in data interpretation and research outcome, especially for experiments designed for high-throughput genomics and proteomics technologies [15, 16]. No standard criteria or systematic method is currently available to evaluate the purity or cellular composition of microvessels, although it can be detected by visual inspection using light microscopy.

Several pre-analytical and analytical challenges are involved in the development of BrMV assessment, including the small amounts of samples available for analysis, normalizing the amounts of different BrMV samples for comparative quantification, and large variations in purity and cellular composition among different samples. Real-time quantitative reverse transcription PCR (real-time qRT-PCR) is considered one of the most sensitive, accurate and reproducible techniques with a broad dynamic range and high specificity to evaluate gene expression patterns by measuring the relative abundance of mRNA transcripts and to validate data obtained by other methods like cDNA microarrays and RNA-seq[17]. Moreover, utilization of qRT-PCR may take the advantages of unique gene expression patterns among different major cell types in the brain to determine relative purity or cellular composition with the need of diminutive portion of individual BrMV samples. However, the accuracy of real-time qRT-PCR relies upon a suitable reference gene (RG) whose expression levels must remain stable across various cell types within BBB isolates, i.e. all components of the neurovascular unit. Moreover, the reliability of purity measurements requires the identification of proper marker genes (MG) whose mRNA levels are sensitive and linearly associated with the amounts of BrEC and none-BrEC cells in BrMV samples.

The overall aims of this study were to identify suitable RG(s) for use in purity evaluation and gene expression studies on brain microvessels and capillary-depleted brain (CDB) tissues, and to determine the proper MG(s) for relative purity assessment. Given that the expressions of some commonly used RGs are quite different among tissues or under different physiological and pathological conditions[18–21], we investigated by Taqman real-time qRT-PCR method five housekeeping RG candidates which have diverse expression levels and have been reported by others as most stable RGs in different studies of rodent brain tissues[22]. Among four commonly used and comparable methods[23, 24], we utilized GeNorm[25] and BestKeeper[26] algorithms for data analysis toward RG stability evaluation. Alpha-L-iduronidase gene (*Idua*) was included as a Gene of Interest (GOI) to validate the impact of RG selection on normalization of gene expression data using BrMV derived from mice with the genotype of *Idua*^+/+^ or *Idua*^+/-^. For purity assessment, we compared four MG candidates that are highly expressed on BBB-forming brain endothelial cells [7, 27–29], including glucose transporter 1 (*Slc2a1*), claudin-5 (*Cldn5*), cluster of differentiation 31 (also called platelet endothelial cell adhesion molecule, *Pecam1*) and the panendothelial cell antigen *Meca* 32 (also called plasmalemma vesicle-associated protein, *Plvap*). Development of a multiplex real-time qPCR assay has made it possible to simultaneously detect MG and RG in the same reaction. Importantly, the cellular composition of BrMV samples were evaluated by immunofluorescent analysis using specific markers for BrEC, neurons, astrocytes, pericytes, and microglial/brain macrophages. The correlation between the relative purity quantified by real-time qRT-PCR using optimal RG and the purity derived from fluorescent microscopy analyses was further determined. The results identify *Cldn5* and *Pecam1* as most sensitive and reliable marker genes for purity evaluation and *Plvap* as indicator gene for the presence of fenestrated vessels.

## Materials and methods

### Cell culture

As reference for the qRT-PCR analyses, 3T3 cell lines were purchased from the American Type Culture Collection (ATCC, Manassas, VA, USA) and maintained as recommended. Bone marrow derived endothelial cells (BMEC) were obtained from Dr. Yi Zheng (Cincinnati Children’s Hospital Medical Center) and maintained with mEPOC medium with EPOC supplement (US Biological, Salem, MA). The bEnd3 cells were purchased from ATCC and cultured as recommended in DMEM medium.

### Mice maintenance

Mice knock-out for α-L-iduronidase gene on C57BL/6J strain background (*Idua*^−/−^), a mouse model of Hurler syndrome (a neurological lysosomal storage disease), were obtained from Jackson Laboratory (Bar Harbor, ME), in-house bred and genotyped as previously described [30]. The experimental groups were generated using heterozygous male and females (*Idua*^+/−^, HET) as breeding pairs in a pathogen-free facility (with micro-isolator) at Cincinnati Children's Research Foundation (CCRF) in the vivarium fully accredited by the Association for the Assessment and Accreditation of Laboratory Animal Care (AAALAC). All experimental procedures were performed according to the NIH Guidelines for the Care and Use of Laboratory Animals and were approved by the Institutional Animal Care and Use Committee at CCRF.

### Brain collection and microvessel isolation

Transcardiac perfusion was employed to purge blood vessels and remove residual blood from anesthetized animals as described previously [31]. Under deep anesthesia with sodium pentobarbital (I.P. injection of 40mg/Kg /mouse), each mouse underwent transcardiac perfusion with sterile, ice-cold 1X PBS for at least 3 min. The success of this procedure was confirmed by a loss of color in the liver and the blood vessels that flank the midline of the rib cage. The brain was then quickly obtained from each mouse and dissected for isolation of the cerebrum portion.

Brain microvessels were isolated as previously reported with modifications [32, 33]. Briefly, 10–12 cerebrums from wild type or heterozygous mice were isolated and homogenized in stock buffer (25mM HEPES, 1% dextran in minimum essential medium) on ice using Teflon pestle glassware that have been coated with 1% BSA in PBS for 2 hours to reduce the adhesiveness of microvessels. After homogenization, the even mixture was filtered through 200-μm and 100-μm nylon meshes (Fisher Scientific, Pittsburg, PA), and followed by dextran (20%) gradient centrifugation at 3500 g for 15 min at 4°C. The pellet was resuspended in stock buffer and filtered through a 25-μm nylon mesh (Biodesign Inc., Carmel, NY). The supernatant was vigorously vortexed, and followed by collection of capillary-depleted brain samples (CDB). The microvessels retained by the 25-μm nylon mesh were collected as BrMV samples.

### RNA extraction and reverse transcription

Total RNA was extracted from samples by the combination of TRIzol (Invitrogen, Carlsbad, CA) and Qiagen RNeasy Mini Kit *per* the manufacturer’s instructions. The amount and purity of RNA were measured by UV spectrometry NanoDrop (NanoDrop ND1000, Thermo Scientific, USA). The absorbance ratio at OD260/280 was between 2.0–2.1, and the ratio of OD260/230 was around 2.0. Extracted total RNA was reverse transcribed at a concentration of 25 ng/μL using High Capacity cDNA Reverse Transcription Kit (Applied Biosystems, USA) according to the manufacturer’s instructions.

### Real-time quantitative PCR

The Taqman primer/probe sets for cDNA of selected reference genes and marker genes were either designed using Primer Express^TM^ software (Version 1.5, ABI Prism) or ordered from Applied Biosystems (ABI, Carlsbad, CA) (Table 1). All real-time qPCRs were performed on 96-well PCR plates with the ABI7900 Real-Time PCR System using TAQMAN PCR MASTER MIX (ABI) in a 20-μL reaction volume. The PCR amplification condition included 2 min at 50 ^o^C, 10 min at 95 ^o^C, followed by 40 cycles of 15 s at 95 ^o^C and 1 min at 60 ^o^C. For each biological sample, the PCR was performed at duplicate for three times. GraphPad Prism V5 (GraphPad Software, Inc.) was used to create the box-and-whisker plots. For each gene in a given condition, the average CT values of all biological replicates corresponding to the samples were pooled together. Boxes correspond to CT values within the 25^th^ and 75^th^ percentiles and the median is represented by a horizontal line. Whiskers include CT values within the 10^th^ and the 90^th^ percentiles and CT values outside this range (outliers) are represented as dots.

**Table 1:** Real-time RT-qPCR designs for RG, MG candidates and GOI with expression levels.

Gene symbol (name)	Function/Primer and Probe (source)	CT value range *	Amplicon size (bp)	R^2^ **
**Reference gene (RG)**				
***Actb*** (beta-actin) (self-designed)	Cytoskeletal structural protein F:GCTTCTTTGCAGCTCCTTCGT R:CCAGCGCAGCGATATCG Probe: CACCAGTTCGCCATGG	14–17	75	0.988
***Gapdh*** (glyceraldehyde 3-phosphate dehydrogenase)	Glycolytic enzyme Mm99999915_q1*(ABI)	15–17	107	0.998
***Pgk1*** (phosphoglycerate kinase 1)	Glycolytic enzyme Mm00435617 m1 (ABI)	18–20	137	0.999
***Tbp*** **(**TATA-box binding protein)	General RNA polymerase II transcription factor Mm00446973 m1 (ABI)	21–23	73	0.991
***Hmbs*** (hydroxymethylbilane synthase)	Enzyme of the heme biosynthetic pathway Mm01143545-m1 (ABI)	22–24	81	0.986
**Marker gene (MG)**				
***Slc2a1*** (Glucose transporter 1)	Glucose transporter Mm00441473_m1 (ABI)	15–17	62	0.968
***Cldn5*** (Claudin 5)	Integral membrane protein Mm00727012_s1 (ABI)	17–18	82	0.969
***Pecam1*** (CD31, or platelet endothelial cell adhesion molecule)	Leukocyte migration, angiogenesis and integrin activation, Mm01242584_m1 (ABI)	19–20	71	0.968
***Plvap*** (plasmalemma vesicle associated protein, or MECA32)	Endothelial cell marker Mm00453379_m1 (ABI)	25–28	71	0.971
**Gene of interest (GOI)**				
***Idua*** (alpha-L-iduronidase) (self-designed)	Lysosomal enzyme F:CTGATTTTGGTCTGGTCAG R:CTGGGCTGAACACAAAGAGG Probe: TCCAAGTGCCTGTGGAC	24–26	139	0.998

* The Ct value ranges were derived from RT-qPCR of cDNA product from 10–50 ng RNA in endothelia cell lines and brain isolated (BrMV and CDB) for RGs, or 20–50 ng RNA in BrMV for MG and GOI.

** correlation co-efficiency (r^2^) for each candidate gene was obtained from standard curves using serial dilution of CDB samples.

### Immunofluorescent analyses

To determine cellular composition of brain microvessels, freshly isolated BrMV samples were centrifuged at 2000 rpm for 5 min in a Cytospin^TM^ 4 Centrifuge, followed by fixation with 4% (w/v) paraformaldehyde. The slides were then stained with fluorescein-labeled Lycopersicon esculentum lectin (in the brain, only endothelial cells are positive for lectin) (Vector Laboratories, Burlingame, CA) and/or antibodies against NeuN (neuronal marker; Cell Signaling Technology, Danvers, MA), GFAP (astrocyte marker; Abcam, Cambridge, MA), CD68 (microglial marker; Fisher Scientific, Pittsburgh, PA), or PDGFR-β (pericyte marker; Santa Cruz Biotechnology, Santa Cruz, CA). Slides were mounted using Vectashield mounting medium with DAPI (Vector Laboratories, Inc.) and observed with an inverted fluorescence microscope (Nikon Eclipse Ti-E, Nukon Instruments Inc., USA). For quantitative analysis, each BrMV sample was evaluated with a total of > 500 nuclei counted from more than 20 views.

### RG data analyses

To select a suitable reference gene, the mRNA expression levels of individual reference genes were statistically analyzed with two different software programs, geNormPLUS [25] and BestKeeper [26], according to manufacturers’ instructions. The geNormPLUS algorithm determines the stability measure (M) of the internal control gene as the average pair-wise variation of each reference gene with all other reference genes, resulting in a ranking of the most stable genes. The lower the M value, the higher the gene stability; a good reference gene should have an M below 0.5 in homogeneous sample sets [25]. BestKeeper calculates standard deviations (SD) and the coefficient of variance (CV) based on the Ct values of all candidates, the higher the SD, the lower the gene stability[26].

### Statistical evaluation

Quantitative assays were performed in duplicate or triplicate from at least two individual experiments. Data are presented as mean ± standard deviation (SD) unless specified. Comparisons between two groups were performed using two-tailed Student t-tests unless specified. P-values of lower than 0.05 were considered as statistically significant.

## Results

### Selection of candidate reference genes and reproducibility of real-time RT-qPCR

Five commonly used housekeeping genes were selected from different cellular function groups, including *Gapdh*, *Actb*, *Tbp*, *Hmbs* and *Pgk1*, which have diverse expression abundance (over ~2-log fold) (Table 1). Taqman-based qPCR was employed to increase assay specificity and minimize any quantitative contribution from genomic DNA contaminants. The intra-assay coefficient of variation for Ct values was derived from 3 PCR reactions each in duplicate from the same reverse transcription (RT) product, ranging from 1.1% for *Tbp* to 2.2% for *Gapdh* (S1 Table). The inter-assay variation determined by 9 PCR repeats using 3 RT products varied from 1.2% for *Tbp* and *Hmbs* to 1.8% for *Pgk1*, suggesting consistently low systematic errors for all RT-qPCR settings. The difference between mean Ct values from individual RT products and those from 3 RT products was minimal (<2.06%). The data validate real-time RT-qPCR systems for all RG candidates tested with high accuracy and reproducibility.

### Evaluation of expression stability among RG candidates in endothelial cell lines and brain samples

**Fig 1:**
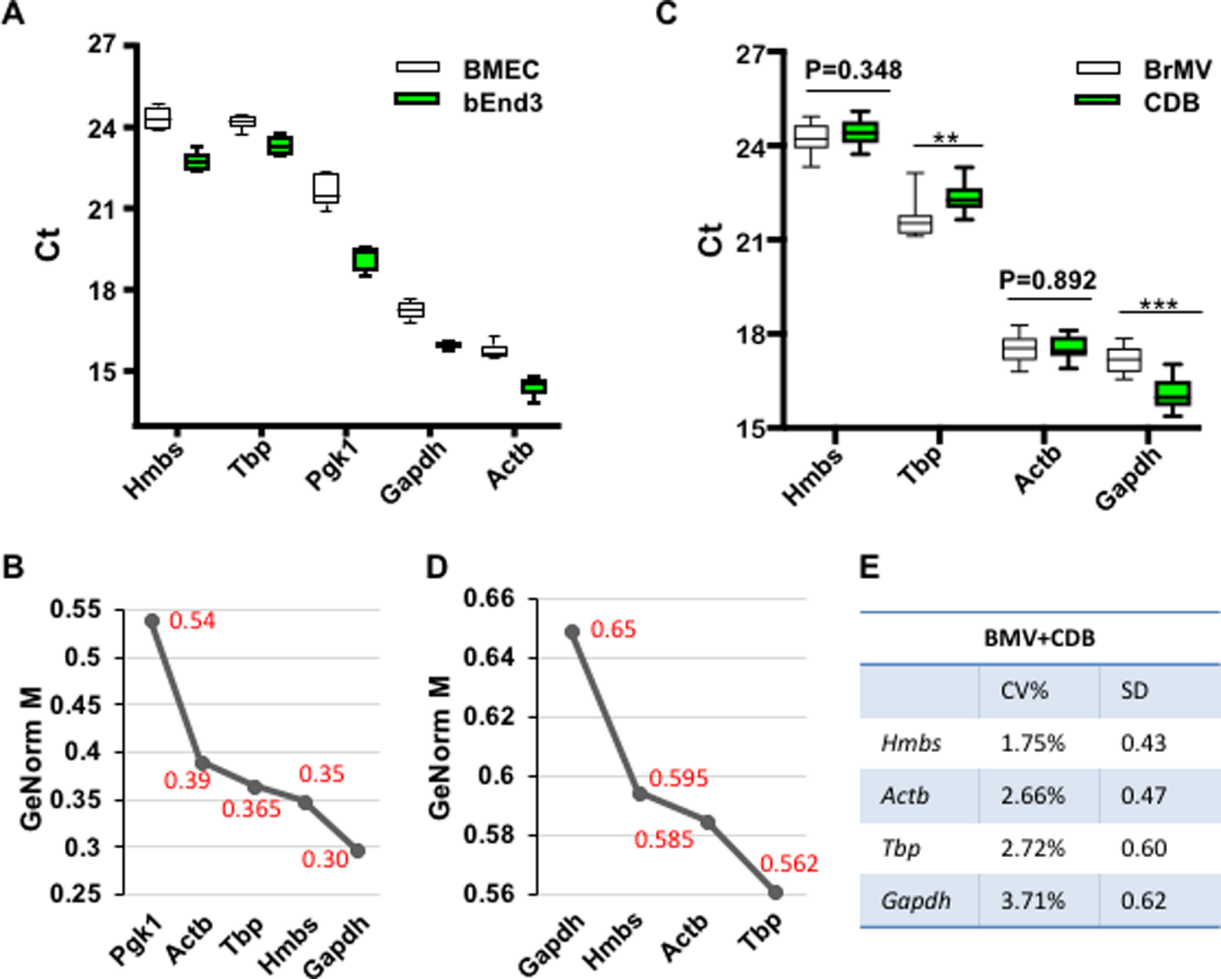
The expression stability of RG candidates among endothelial cell lines and brain samples. Total RNA was extracted and converted into cDNA by reverse-transcription at 25 ng/ul, and followed by real-time qPCR with 25 ng/reaction. (A) Distribution of cycle threshold (Ct) values for five RG candidates by quantitative RT-PCR in BMEC and bEnd3 cell lines. The experiments were repeated 3 times in duplicate reactions. Boxes showed the range of Ct values for each candidate gene. The central line indicated the median Ct; the extended upper and lower indicate 75 and 25 percentiles. (B) The average expression stability (M value) of RG candidates in two endothelial cell lines analyzed by geNorm. RG candidates were ranked from the least stable to the most stable (left to right). (C) Ct values for four RG candidates in BrMV and CDB. Data were derived from 10 BrMV isolation experiments with 4 from WT mice, 2 from Het and 4 from *Idua* knock-out mice. Each sample was tested 3 times in duplicate. **, p<0.01, and ***, p<0.001. (D) The expression stability of four reference genes among all BrMV and CDB samples analyzed by geNorm. (E) RG candidates were ranked in the order of their expression stability evaluated by Bestkeeper based on coefficient of variation (CV%) and SD.

To identify the optimal RG(s) that are steadily expressed among BBB-forming endothelial cells and non-endothelia CNS cells, we quantified candidate gene expression by real-time RT-qPCR using endothelial cell lines and brain isolates (Fig 1). The mRNA levels for all five RGs were significantly higher in mouse cerebral endothelial cell line bEnd3 (representing BrMV) than those in bone marrow derived endothelial cells (BMEC, representing regular endothelial cells), with the mean Ct values ranging from 15.1 to 24.3 (Fig 1A). *Gapdh* and *Actb* genes were most abundantly expressed (mean of Ct at ~15), and followed by *Pgk1* (~20), *Hmbs* and *Tbp* (23–24). The average gene stability measure (M) was calculated using GeNorm software, showing that *Pgk1* gene has the highest M value (>0.5) and is thus the most unstable reference gene (Fig 1B). We excluded *Pgk1* from the candidate pool in subsequent evaluations.

To include variations contributed by the purity of different BrMV isolates and by the physiological condition of the brain, we isolated 10 pairs of brain samples (BrMV and CDB), including 4 samples from normal C57/Bl6 mice (WT), 4 samples from mice with a neurological lysosomal storage disease (Hurler syndrome, *Idua*-deficient)[34] and 2 samples from heterozygotes for *Idua* (Het). The qualities of RNA samples were verified by gel electrophoresis, showing sharp and intense 28S and 18S rRNA bands (with density ratio of 28S/18S >1.0 for all samples) (S1 Fig). The mRNA levels of the remaining 4 house-keeping genes were similar in BrMV and CDB for *Actb* (p = 0.892) and *Hmbs* (p = 0.348), and significantly different for *Gapdh* and *Tbp* (p<0.01) (Fig 1C). Four reference genes displayed a wide expression abundance, with mean Ct values ranging from 16 to 24 (with rank of *Gapdh*>*Actb*>*Tbp*>*Hmbs*). Based on GeNorm analysis for pairwise variations, the RGs in all BrMV and CDB samples were ranked from more stably expressed to less stable ones as *Tbp*>*Actb*>*Hmbs*>*Gapdh* (Fig 1D). We also analyzed the mRNA stability of RGs using the BestKeeper algorithm, which creates a “best keeper” gene rank based on the geometric mean of each candidate’s raw Ct values (Fig 1E). After comparing standard deviation (SD), coefficient of variation (CV), the coefficient of correction (Y) and P-value of all reference genes in BrMV and CDB, the rank from the most stable to least stable were *Hmbs*>*Actb*>*Tbp*> *Gapdh*. Combining the ranking results from GeNorm and Bestkeeper as well as stabilities between BrMV and CDB, *Actb* and *Hmbs* would be the best reference genes for BrMV samples. These results suggest that the expressions of housekeeping genes may vary significantly dependent upon cell types, and validation of stable expression of RG(s) among relevant samples would be necessary to enhance repeatability and accuracy.

### The effect of choosing standard samples and reference genes on the accuracy of RNA quantification

**Fig 2:**
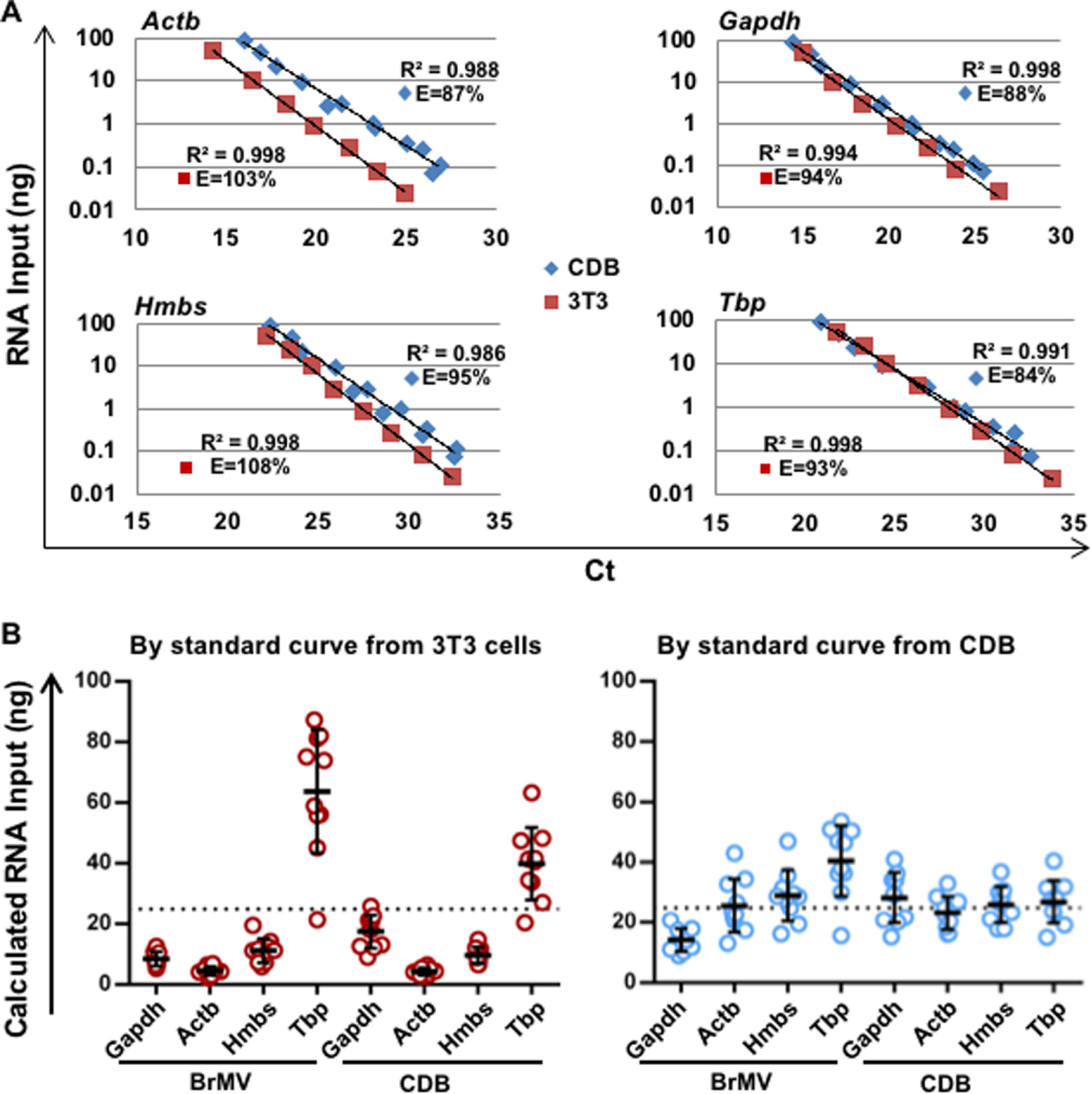
Quantification of RNA input using RG standard curves derived from 3T3 or primary CDB samples. Total RNA samples isolated from 3T3 cell line or CDB samples of C57/Bl6 mice were serially diluted, applied for reverse transcription, and followed by qPCR of 4 reference genes. (A) Standard curves of RNA amounts generated by qPCR of 4 RGs. Data were derived from 3 dilution sets with each amplified in triplicates. E, amplification efficiency for a combination of RT and qPCR steps calculated from the slope of each standard curve; R^2^ range from 0.986 to 0.998. (B) Quantification of total RNA inputs of BrMV and CDB isolates from 10 isolation experiments using different standard curves. Reverse transcription was conducted at 25 ng/ul (by NanoDrop) for all RNA samples, and real-time qPCR was performed using 25 ng/reaction (and indicated as dashed line). Each symbol represents mean of calculated RNA amount derived from Ct value of triplicate qPCR reactions of one sample. Short lines represent mean ± SD of RNA amounts calculated using different standard curves from each of RGs.

Reliable and accurate measurement of reference gene expression is critical for normalization of RNA input among assays/samples toward quantification of target gene expression regardless of using relative (fold changes) or absolute (copy number) methods. To determine if and how the choice of standard samples and internal reference genes would affect mRNA quantification, we compared standard curves of RG candidates using serially-diluted mRNA from either NIH3T3 cells or CDB samples isolated from WT mice (Fig 2A). The figure shows different separations of standard curves between two standard sample types among 4 RGs, with the most dissimilarity for *Actb* and the least for *Tbp*, suggesting relatively more abundance of all reference genes (except for *Tbp*) in 3T3 than in CDB samples. The RT-qPCR efficiencies calculated using slopes of standard curves in 3T3 cells ranged from 93% to 108% (with R^2^ ranging from 0.994 to 0.998), validating primer/probe amplification efficiencies. Less efficiencies were detected in isolated CDB samples (ranging from 84% to 95%), most likely resulted from reagents and procedures involved in multistep sample preparation that may reduce reverse-transcription and/or qPCR efficiencies. Fig 2B shows how the quantification of mRNA input of BrMV and CDB samples can be affected by using different standard curves. When using 3T3-based curves (left panel), the mRNA amounts of all samples were either significantly over-estimated with *Tbp* as RG or under-estimated with remaining 3 reference genes, even though the same amounts of RNA (25 ng) were used as determined by NanoDrop. The quantification of RNA amounts of brain samples was much more consistent when calculated by CDB-derived standard curves (right panel), with *Actb* and *Hmbs* presenting most comparable measurements in both BrMV and CDB. These results demonstrate that the standard curves derived from CDB samples are more appropriate for calculating RG expression, suggesting that proper choice of standard samples (tissue/cell types) to generate standard curves would affect the accuracy of target expression evaluation. The data also indicate that *Actb* and *Hmbs* are the most reliable reference genes for mRNA normalization in BrMV and CDB isolated.

### Validation of *Actb* as the best reference gene by IDUA expression in BrMV

**Fig 3:**
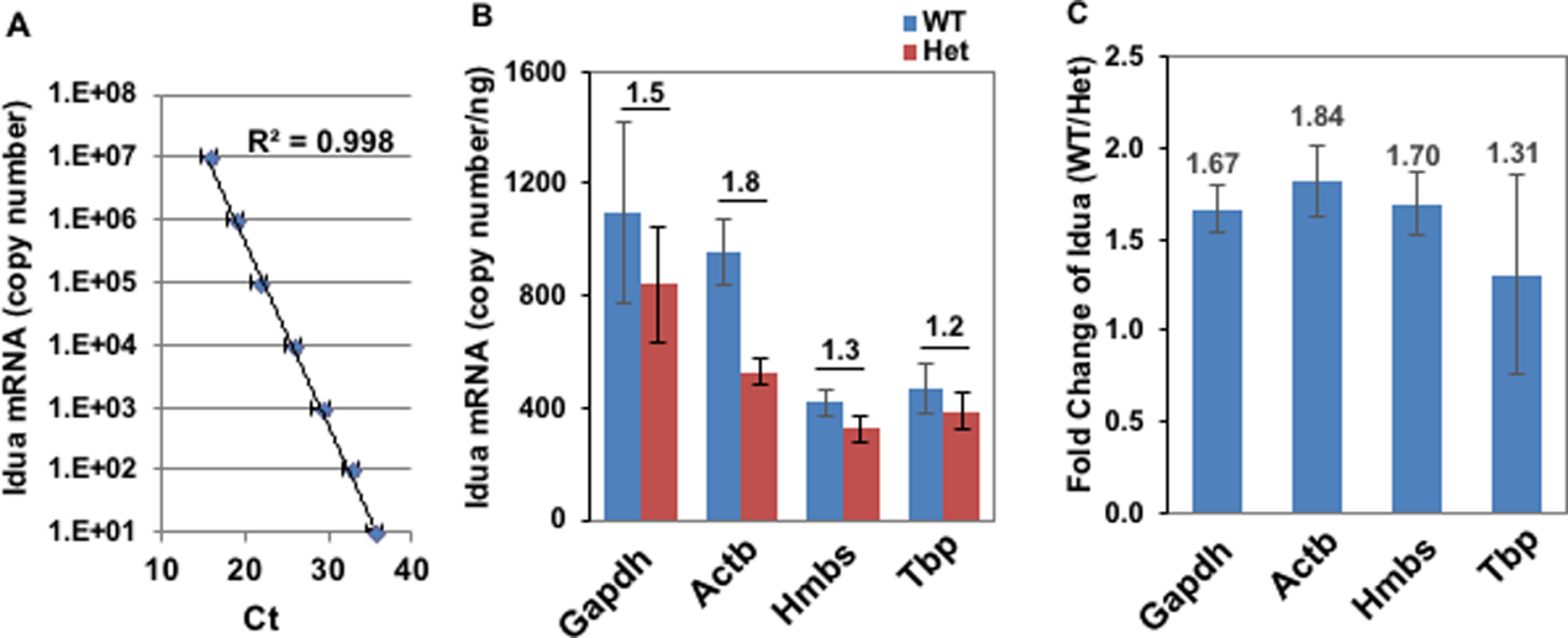
Verification of *Actb* as the best reference gene by *Idua* expression in BrMV of WT mice and Het mice. **(A)** Standard curve for absolute quantification of *Idua* mRNA. A plasmid containing *Idua* cDNA was used for generating standard curve with copy numbers by qPCR. Data was derived from 2 sets of standard samples, each amplified three times in duplicate. Error bars, standard deviation. (B, C) *Idua* expression in BrMV isolated from either wild-type C57/Bl6 mice (WT) or littermates of heterozygous for *Idua* knock-out (Het) with normalization by RG candidates. Total RNA from 4 WT and 4 Het samples were examined by RT-qPCR and calculated either by absolute *Idua* standard curve for copy numbers per ng RNA (B), or by ΔΔCt method for relative *Idua* fold changes (C).

Based on analyses of a cluster of samples with GeNorm/BestKeeper and RT-qPCR amplification efficiency (i.e. standard curves), *Actb* and *Hmbs* were considered the most stable reference genes for quantitative studies with primary BrMV and CDB samples. To further verify the observation, the mRNA expression levels of *Idua*, a lysosomal enzyme, were measured as a gene of interest in BrMV and CDB isolated from either wild type C57/Bl6 mice or littermates that contain a *Idua*-knockout allele (heterozygotes) (Fig 3). The transgenic IDUA knockout murine model (MPS I), generated by disruption of the open reading frame with an insertion in exon 6, exhibits no detectable levels of *Idua* mRNA or enzyme activity[35]. First, we generated a standard curve for *Idua* copy number using a plasmid containing *Idua* cDNA (Fig 3A). Then we measured threshold values of *Idua* mRNA by RT-qPCR in BrMV samples (Fig 3B and 3C). When using absolute quantification with analysis for mRNA copy number (Fig 3B), *Actb* leads to near-expected ratio (1.8) between WT and HET (expected 2), followed by *Gapdh* (1.5), *Hmbs* (1.3), and *Tbp* (1.2). When analyzing data with ΔΔCt methods for relative quantification of fold changes between sample sets (Fig 3C), *Actb* results in near-expected ratio (1.84), followed by *Hmbs* (1.70), *Gapdh* (1.67), and *Tbp* (1.31). These results show that expression profile of GOI in isolated BrMV samples can be highly affected by the choice of RG, validating *Actb* as the most suitable RG for sample normalization regardless of quantification analysis used (while *Hmbs* may also be suitable for relative quantification).

### Choice of marker genes for purity determination in BrMV samples

**Fig 4:**
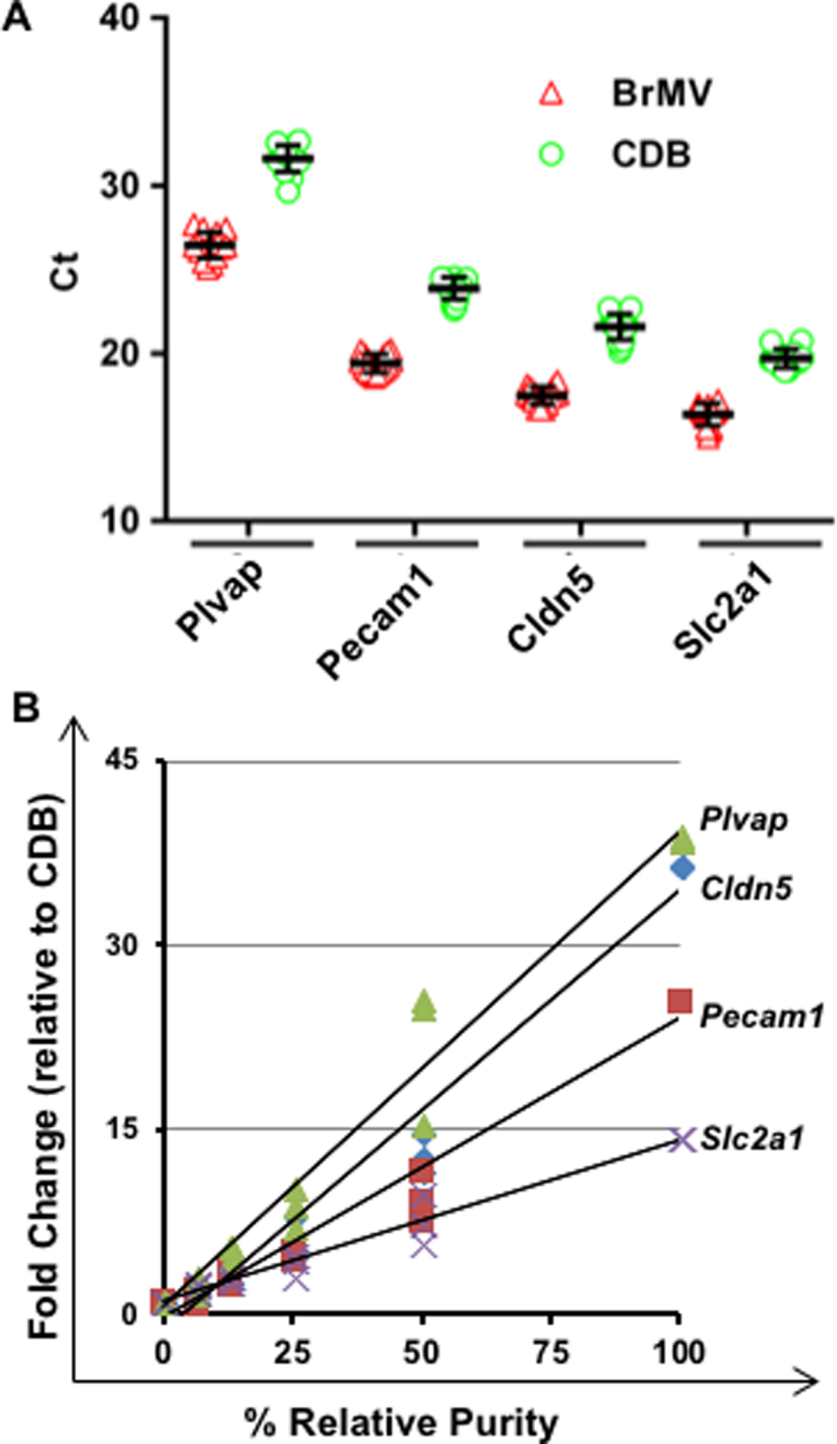
Choice of marker genes to assess the purity of BrMV samples. (A) Distribution of threshold cycle (Ct) values for MG candidates in BrMV and CDB samples by RT-qPCR. Each sample is repeated 3 times in duplicate reactions, and each symbol represents the mean of one sample. Short lines represent mean ± SD of Ct values for each marker gene, n = 10. (B) Relative purity curves determined by relative quantitation of mRNA between different marker genes and *Actb* (as reference gene). One BrMV sample with relatively high marker gene expression over CDB samples was designated as “100%” relative purity and two sets of standard samples were generated by serial dilution of this BrMV with CDB samples (considered as “0%”). Data were derived from 2 sets of standard samples with 3 RT-qPCR experiments in duplicate reactions. The R^2^ ranges from 0.968 to 0.971.

To identify appropriate marker genes for evaluation of the purity in BrMV samples, we selected several candidate genes that are highly expressed in BBB-forming brain endothelial cells, including *Slc2a1*, *Cldn5*, *Pecam1* and *Plvap*. Fig 4A shows expression variation of different marker genes in BrMV and CDB samples, with *Slc2a1* mRNA as the most abundant MG and followed by *Cldn5*, *Pecam1* and *Plvap*. It also verifies that the mRNA levels of four MGs are significantly elevated in BrMV comparing to capillary-depleted brain samples (i.e., CDB). Fig 4B exhibited standard curves of relative purity for BrMV samples based on relative quantification (fold changes) of mRNA levels of different marker genes with *Actb* as the reference gene. The standard samples were generated by serial dilution of RNA from a BrMV sample expressing relatively high levels of MGs (designated as 100%) with RNA from CDB samples (0%) at different percentages. The increase in mRNA levels for all selected marker genes were linearly associated with relative purities in BrMV standard samples. *Plvap* displays the highest elevation in BrMV compared to non-vascular brain samples that expressed barely detectable levels of *Plvap*.

**Fig 5:**
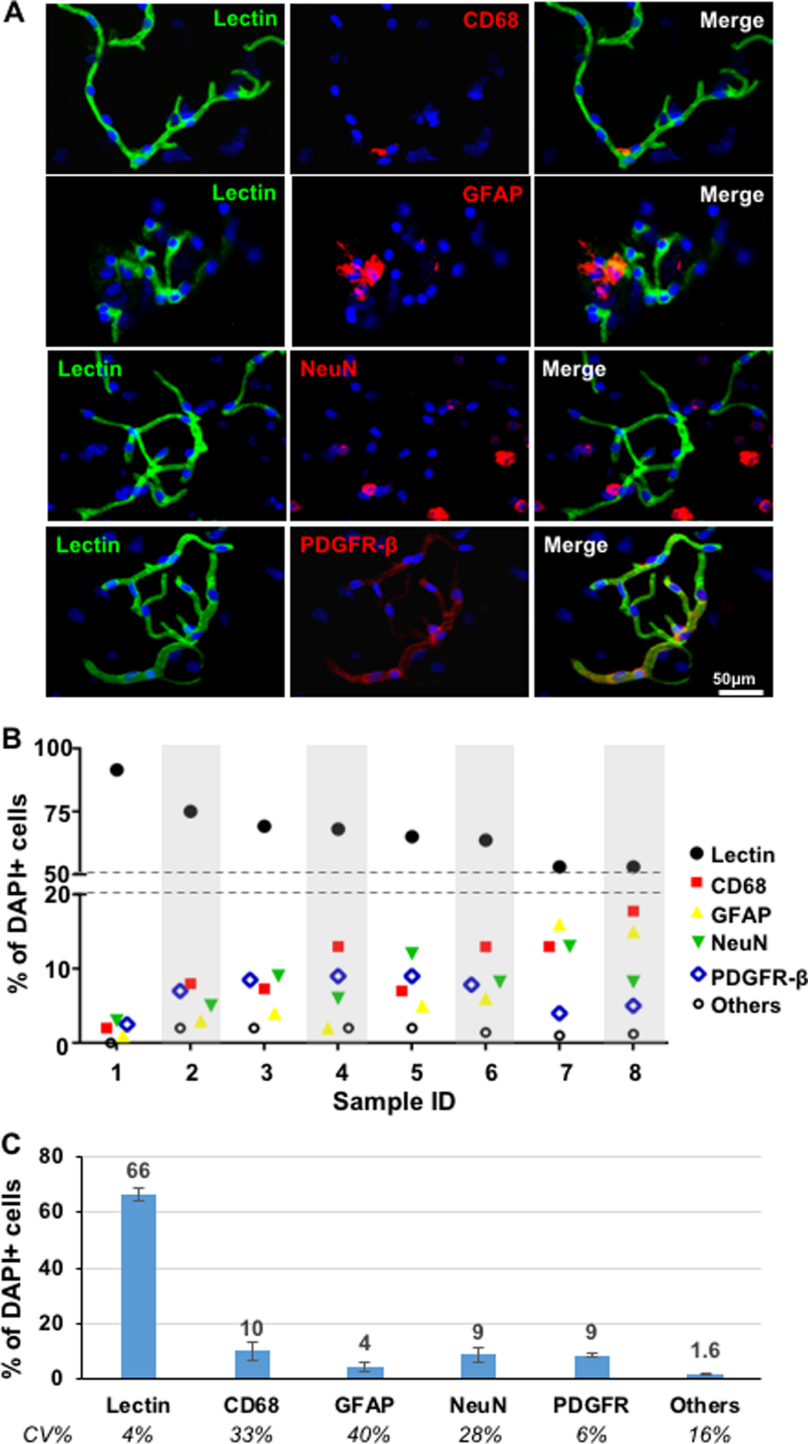
Cellular components of BrMV isolates determined by immunostaining. (A) Representative pictures of immunofluorescence analysis for BrMV isolates. The brain BrMV isolates were stained with fluorescein-labeled (green) lectin for BBB-forming endothelial cells, and Alexa 568-conjugated (red) anti-mouse CD68 for brain macrophages, anti-mouse GFAP for astrocytes, anti-mouse NeuN for neurons, or anti-mouse PDGFR-β for pericytes, and followed by counter-staining with DAPI (blue) for nucleic. Scale bar represents 50 μm for all views. (B) Semi-quantifications of cellular composition for nine BrMV samples. Each sample was evaluated with a total of >500 nuclei. Pearson correlation coefficient between Lectin^+^ and other cell types are -0.862 for CD68^+^ cells, -0.837 for GFAP^+^ cells, -0.776 for NeuN^+^ and -0.190 for PDGFR+ cells. (C) Semi-quantification of main cell types in BrMV isolates. Data are derived from 4 BrMV samples with similar purities (ranging of 64–69%) as determined by immunostaining; error bars represent SD. CV, coefficient of variation.

To evaluate cellular composition in BrMV isolates, we conducted immunofluorescence microscopy analysis in the isolates to determine proportional contributions from BBB-forming brain endothelial cells as well as other brain parenchymal cell types, including brain macrophages, astrocytes, neurons and pericytes (Fig 5). Representative views are shown in Fig 5A, and semi-quantifications for nine isolated samples are shown in Fig 5B. The BrMV isolates mainly contain endothelial cells as indicated by lectin^+^ staining[36], varying from 53% to 92%. Moreover, the reduction of purity (lectin^+^%) among samples are mostly associated with increasing amounts of microglia/brain macrophages (Pearson correlation coefficient of -0.862), astrocytes (-0.837) and neurons (-0.776). When evaluating BrMV isolates with similar purities (at 64–69%) as shown in Fig 5C, PDGFR-β^+^ pericytes have 9% cellular contribution with the least changes among samples (6% for CV), followed by similar amounts of CD68^+^ (a lysosomal marker indicative of phagocytic activity in microglia) brain macrophages (mean of 10%) and NeuN^+^ neurons (mean of 9%) that varied substantially among samples (CVs of 33% and 28%). The GFAP^+^ astrocytes were least abundant components (4%) with minimal unknown-type of cells (1.6%). The results validate the isolation of brain capillary microvessels, with BrEC comprising the majority of cells, and with cellular contaminants mostly from pericytes, neurons and brain macrophages (i.e., perivascular microglial).

### Correlation analyses for selection of optimal purity marker genes

**Fig 6:**
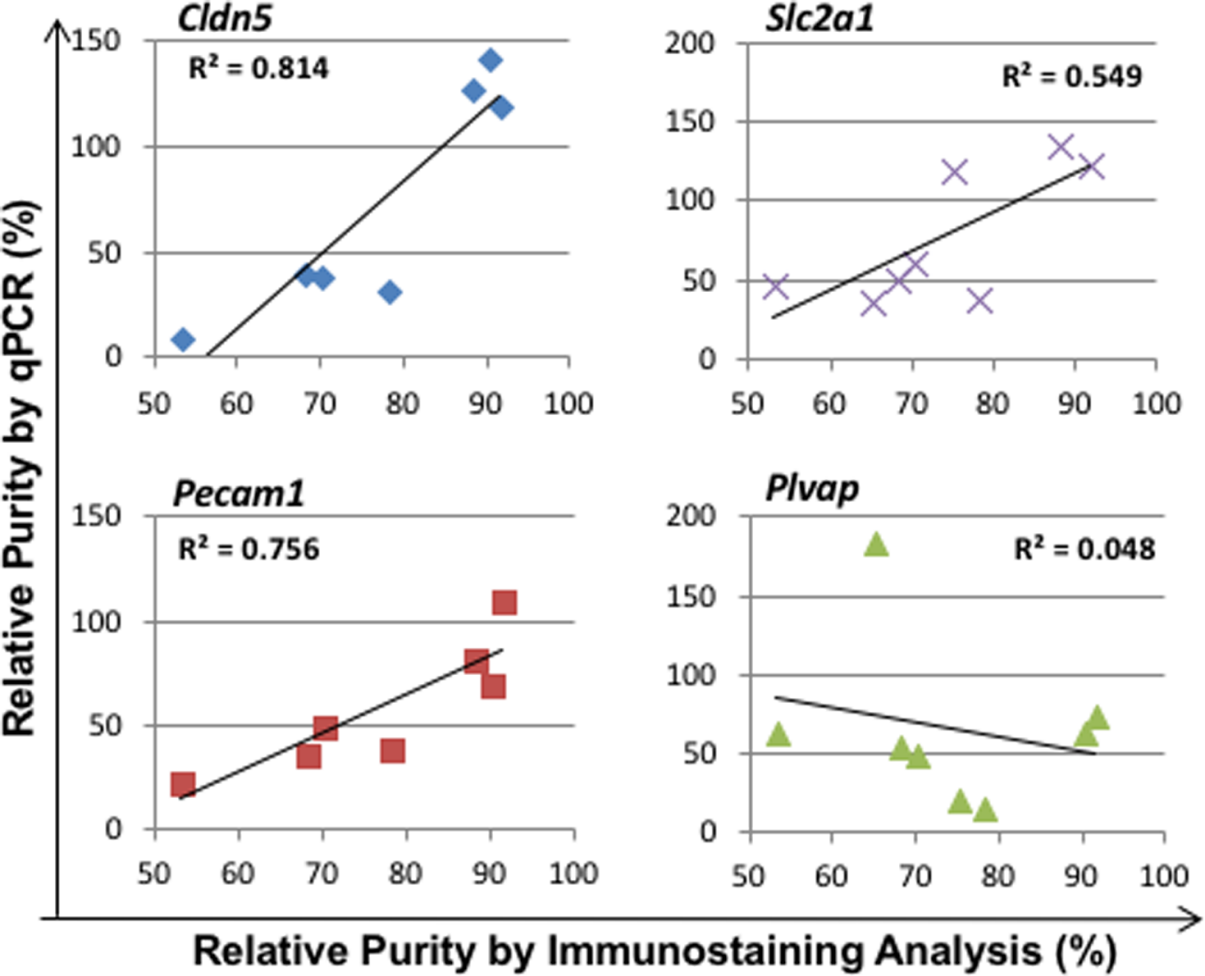
Correlation of purities measured by both qPCR and immunofluorescent microscopy. The relative purities of BrMV samples were determined by real-time RT-qPCR with relative comparison of mRNA abundances using standard curves of different marker genes as described in Fig 4, as well as by immunofluorescence analysis as described in Fig 5. Each symbol represents relative purity of individual BrMV sample derived from 3 RT-qPCR experiments in duplicate reactions for qPCR, as well as from evaluation of >200 DAPI^+^ nuclei from cytospin slides by immunostaining analysis.

The relative purities from BrMV isolates were calculated using standard curves derived from RT-qPCR with 4 marker genes as shown in Fig 4. BrMV samples with relatively high numbers of BrEC (>50%) were further correlated with immunostaining analyses for the percentages of lectin^+^ cells as shown in Fig 6. The purest BrMV of all samples tested contained 92% BrEC as determined by immunofluorescent analysis, with 0–3% for every other cellular component evaluated (Fig 5B). The relative purities of this BrMV sample calculated by standard curves of MGs were 119% by *Cldn5* (with 41-fold increase over CDB), 111% by *Pecam1* (with 27-fold), 124% by *Slc2a1* (with 17-fold), and 74% by *Plvap* (with 29-fold) as shown in Fig 6. Among four MGs evaluated, *Cldn5*-derived relative purity exhibits the highest correlation with purity measurement derived from immunostaining analyses (R^2^ = 0.814), followed by *Pecam1* (0.756) and *Slc2a1* (0.549). However, *Plvap* expression levels in BrMV samples have no correlation with purity from immunostaining with correlation coefficient as low as 0.048. These results demonstrate that *Cldn5* and *Pecam1* are reliable MGs for purity evaluation of BrEC isolates.

## Discussion

This study is the first to provide a comprehensive evaluation of commonly used reference genes for their reliability in normalization of real-time RT-qPCR, as well as BBB-related marker genes for their suitability in purity assessment of brain micro-vasculatures. Minimal variation in RG expression is a prerequisite for the normalization of RT-qPCR studies to avoid bias or misleading conclusions[37]. However, there is no single perfect reference gene to fit all because identification of valid RGs seems to be associated with investigated tissues, organs, species or experimental conditions[19, 20, 38, 39]. Therefore there have increasing needs and interests to evaluate and validate RGs for studies in different scientific research disciplines, such as microbiology[40, 41], plant science[42–44], cancer research[45, 46], and neuroscience[21, 47, 48]. Dictated by the highly dynamic and complex vascular structure of the brain, contamination of BrMV isolates with other cell types of neurovascular units is inevitable. We have identified *Actb* out of 5 candidates as the most suitable RG that was further validated by quantification of *Idua* mRNA levels in BrMV isolated from mice with one or two expressing alleles. Moreover, four marker genes that are known to have higher mRNA abundance in BrEC than in other brain cell types were evaluated for purity assessment using ΔΔCt algorithm [49] against *Actb* as RG. *Cldn5* and *Pecam1* were identified as most suitable MGs that were linearly associated with the percentages of brain capillary endothelial cells determined by immunofluorescent analysis, and high *Plvap* levels would serve as an indicator for contamination of fenestrated blood vessels.

Based on a quantitative review of the literature (from 2007 to 2015) on choices of RGs, *Actb* (38%), *Gapdh* (38%), and *18S* rRNA (12%) continue to be common RG choices in studies of vertebrate gene expression [22]. However, *18S* rRNA was not selected as a candidate RG in this study for several reasons. First, *18S* rRNA has characters distinguished from a typical protein-producing mRNA, including i) lack of a conventional poly(A) tail, ii) containing complicated 2^nd^ structure and iii) resistance to degradation much more than mRNA. Secondly, it has 300 to 400 copies in the genome and does not contain introns. Therefore, it is more challenging to eliminate quantitative contribution from genomic DNA contamination. Thirdly, rRNA is much more abundant (several log-fold) than most mRNA transcripts, which makes it less sensitive to changes a reference gene intended to control for. It would also be difficult to detect non-abundant messages while remaining in the exponential phase of amplification for 18S rRNA. To increase the diversity of mRNA abundance among RGs, we selected *Hmbs*, *Tbp* and *Pgk1* which have been reported by others as most stable RGs in different studies of rodent brain tissues[22].

The selection of *Actb* as the optimal RG for BBB study is based on a combination of multiple factors. First, Ct values of *Actb* remained most stable between BrMV and capillary-depleted brain samples (p = 0.892) while all other RGs showed highly or near significant difference. Secondly, studies have shown small variations in the outcomes of data analysis across multiple RG candidates with three commonly used algorithms[23], including GeNorm [25], BestKeeper[26] and NormFinder[50]. We evaluated five RG candidates by GeNorm using relatively homogenous endothelial cell lines derived from either bone marrow or brain, resulting in the removal of PGK from further study due to its higher-than-cutoff M value (>0.5) as previously suggested[51]. Primary samples of BrMV and CDB from 10 isolation experiments derived from difference genotypes (for *Idua* gene) were evaluated by both GeNorm and BestKeeper platforms, with *Actb* consistently ranked as 2^nd^ best stable RG (HMBS ranked 3^rd^ and 1^st^, respectively). Thirdly, the initial RNA inputs of BrMV and CDB samples determined by NanoDrop spectrophotometer were compared to those calculated by CDB-based standard curves derived from each RG candidate whose main role is for normalization of RNA amounts. Consistent matches were observed for *Actb*, while others led to significant over- or under-estimation in BrMV samples. Finally, the suitability of *Actb* to normalize RT-qPCR data for BrMV samples was validated by quantification of *Idua* mRNA copy numbers in brain samples isolated from WT (*Idua*^+/+^) and heterozygous (*Idua*^+/-^) mice (for expected ratio of 2). Taken together, we have utilized different approaches that identified *Actb*, one of the two non-muscle cytoskeletal actins[52], as the most suitable RG for studies of brain microvessels. *Hmbs* may serve as the 2^nd^ RG for relative quantification (via ΔΔCt method).

Several aspects of assay design may influence the outcome of RT-qPCR based experiments. First, varying degrees of RNA fragmentation among samples may affect the accuracy of quantification, especially when post-mortem samples or micro-dissecting samples are involved. Determination of RNA integrity number (RIN) is often suggested for each sample, which can provide quantitative measure for RNA quality [53]. Alternatively, the negative impact from poor qualities of hard-to-get samples can be minimized by utilization of internal RG amplification simultaneously together with the gene of interest in multiplex reactions, as well as relative quantification using ΔΔCt methods[54, 55]. In this study, we verified the relative integrity of RNA samples by gel electrophoresis as a quality control measurement. Importantly, we ensured that amplicons of all qPCR assays were less than 140 base pairs since it has been reported that short amplicons (below 250 bp) are much less dependent upon RIN values in qPCR-based assays[54], even when using RNA samples from human post-mortem brain with RIN ranging from 5.9 to 9.9 [56]. Secondly, the choice of proper standard samples is critical in RG determination and quantification of GOI expression regardless of relative comparison (using ΔΔCt methods) or absolute PCR for mRNA copy numbers out of total RNA. Standard samples are used to generate either amplification efficiency (E with 100% or e with 2 as the perfect score) of each RT-qPCR setting for relative qPCR comparison, or standard curves to determine RNA inputs for quantification of absolute expression levels of the target gene[55]. We conducted a direct comparison using murine 3T3 cells with capillary-depleted brain samples. While utilization of 3T3 cells is convenient, the resulting standard curves led to significant under- or over-estimation of RNA amounts in BrMV and CDB samples with all RG candidates. The data demonstrates the importance of choosing and validating proper standards, ideally using actual samples, for normalization of RT-qPCR analyses. Thirdly, Taqman primer-probes and SYBR Green/EvaGreen are the most commonly used real-time PCR chemistries although many other detection chemistries have also been developed[57, 58]. While being cost-effective and generally applicable for all qPCR reactions, SYBR Green (as a dsDNA intercalating agent) leads to the detection of both specific and non-specific PCR products, and it is necessary to verify absence of non-specific amplification. The Taqman approach provides high specificity for the target sequence and resistance to DNA contamination, which is especially important for quantification of low abundant mRNA GOIs. We found Taqman-qPCR more suitable for the small amounts of BrMV samples, corresponding with observations by others for clinical diagnosis and treatment controls[59]. Finally, confirmative comparison of RG candidates is a critical step by incorporating a GOI with predictable mRNA changes. The quantification of IDUA mRNA in CDB samples from WT and Het mice was decisive in the selection of *Actb* as optimal RG, especially when ranks were not identical among different analytical algorithms.

To develop a method of purity assessment for BrMV samples, we evaluated four marker genes with three highly expressed and one (*Plvap*) selectively expressed in brain endothelial cells. *Cldn5* is one of the main integral membrane proteins that comprise BBB tight junctions, with the greatest density in the capillaries and smaller venules, and least in the larger venules[60]. *Pecam1* is normally found on endothelial cells and several types of blood cells, and has been used as a marker for the normalization of protein quantification in endothelial cells[15]. As expected, significantly higher expression of *Cldn5* (mean of 36-fold) and *Pecam1* (mean of 25-fold) were detected in BrMV (with >50% BrEC) than those in CDB samples. Importantly, the relative purity derived from RT-qPCR using both *Cldn5* and *Pecam1* showed a very high correlation (correlation coefficient, r^2^ > 0.81 or 0.76) with % BrEC content quantified by cellular composition analysis using immunofluorescent microscopy. *Slc2a* exhibited the highest mRNA abundance but the smallest difference between BrMV and CDB, showing less coordination in purity evaluation. This is likely due to its broader expression pattern among brain cells (including BrEC, astrocytes and other glia cells)[61], as well as its varying levels among different cell types (high in BrEC and none in neurons) within different BrMV samples. The best isolated BrMV sample in this study contained 92% BrEC with 0–3% for each of other cellular components evaluated. This sample also exhibited 41-fold increase (comparing to CDB) of *Cldn5* levels and 27-fold of *Pecam1*, which corresponded to calculated relative purities of 119% by *Cldn5*, 111% by *Pecam1*. To our knowledge, this is the first platform built to quantify the relative BrMV purity by real-time RT-qPCR. We identified and verified by microscopy analyses that both *Cldn5* and *Pecam1* would serve properly as marker genes for BrMV purity evaluation.

The endothelial cell-specific antigen *Plvap* is normally expressed in peripheral vasculature throughout development, but the expression in the cerebrovasculature is downregulated or ceased upon establishment of the blood-brain barrier[62]. *Plvap*, which is involved in vesicular transport in endothelial cells, is highly expressed in permeable vessels of peripheral tissues, and is upregulated with pericyte loss [63, 64]. It has been used as a robust marker of cerebrovascular inflammation and/or compromised BBB integrity[65, 66]. We investigated the utility of *Plvap* in determination of BrMV purity. Consistent with expectations, near-background levels (Ct > 30) of *Plvap* were observed in CDB samples; while evidently detectable levels were found in BrMV samples that were likely derived from unavoidable contamination of fenestrated vessels of the brain, such as meningeal vessels and those of the choroid plexus. Moreover, almost no correlation was observed between changes of *Plvap* levels and BrEC contents in BrMV samples. On the other hand, one of nine BrMV samples exhibited abnormally high levels of *Plvap*, suggesting the presence of more non-BBB microvessels. Thus, *Plvap* can serve as an indicator gene for abnormally high contamination of fenestrated vessels in BrMV samples.

Simultaneous immunofluorescent analyses for the same set of samples evaluated with relative purity by RT-qPCR has provided conclusive identification of proper marker genes, and elucidated relative quantity of each cellular component within the neurovascular unit. Pericytes, which are vascular mural cells embedded in the basement membrane of blood microvessels[67], stably comprised 9% of cells in BrMV samples with the BrEC component varying from 64% to 69%. Phagocytic brain macrophages or neurons contributed to 10% or 9% of total cells with large sample-to-sample variation. Interestingly, the changes of relative purity among BrMV samples seems to be negatively associated with the amounts of microglia/brain macrophages, astrocytes and neurons, but not so much with pericytes.

## Conclusion

In summary, we have provided a comprehensive study that not only identified and validated *Actb* as the optimal reference gene for quantitative evaluation of brain microvessels (and *Hmbs* as the 2^nd^ RG for relative quantification), but also established a three-MG approach for purity assessment of BrMV samples, i.e., using *Cldn5* and *Pecam1* for BrEC content and *Plvap* for fenestrated microvessel contaminants. The general application of *Actb* as RG for BrMV should be with caution. Validation is recommended for each experimental condition; for example, *Actb* has been found to be downregulated by statins in statin-treated HUVEC cells[68]. Confirmation of RG should use actual samples for evaluation of GOI with known expression levels. Quality evaluation of each RNA sample is needed for both purity (ultraviolet absorbance) and integrity analyses, although quality requirements may vary depending upon the downstream application. BrMV samples with abnormally high levels of *Plvap* (e.g. more than 40-fold over CBD) may not represent high-quality brain microvessels. Depending on the objective of individual study, additional marker genes for various cellular contaminants (pericytes, neurons or brain macrophages/microglia) may be used to pinpoint relative presence of individual component within each BrMV sample. For each study setting, the calculated BrMV purities derived from qPCR of MGs should be adjusted to BrEC contents by other assessments such as immunofluorescent analyses (e.g., 92% BrEC corresponding to 115% purity in this study setting). These methods open the door to more accurate investigations of the BBB for determining changes in physiological and relevant clinical conditions, and for developing RNA-Seq-based molecular atlas of the BBB in animals and humans for new targets, signaling pathways within the neurovascular unit, and new therapies. This study may contribute to the building blocks toward overarching research needs to surmount challenges around the interface between the blood and the brain.

## Supporting information

S1 Fig Examination of RNA integrity and genomic DNA contamination by agarose gel electrophoresis stained with ethidium bromide. (PPTX)

S1 Table Evaluation of repeatability and accuracy of each real-time qPCR setting for reference genes using 3T3 cells. (DOCX)

## References

[1] Zhao Z, Nelson AR, Betsholtz C, Zlokovic BV. Establishment and Dysfunction of the Blood-Brain Barrier. Cell. 2015;163(5):1064–78. doi: 10.1016/j.cell.2015.10.067 ; PubMed Central PMCID: PMC4655822. 26590417 10.1016/j.cell.2015.10.067 PMC4655822

[2] Ochocinska MJ, Zlokovic BV, Searson PC, Crowder AT, Kraig RP, Ljubimova JY, et al NIH workshop report on the trans-agency blood-brain interface workshop 2016: exploring key challenges and opportunities associated with the blood, brain and their interface. Fluids and barriers of the CNS. 2017;14(1):12 doi: 10.1186/s12987-017-0061-6 ; PubMed Central PMCID: PMC5410699. 10.1186/s12987-017-0061-6 PMC5410699 28457227

[3] Pan D. Cell- and gene-based therapeutic approaches for neurological deficits in mucopolysaccharidoses. Curr Pharm Biotechnol. 2011;12(6):884–96. doi: 1389-2010/11$58.00+.00. ; PubMed Central PMCID: PMCPMC4040261. 21235445 10.2174/138920111795542679 PMC4040261

[4] Montagne A, Zhao Z, Zlokovic BV. Alzheimer's disease: A matter of blood-brain barrier dysfunction? J Exp Med. 2017;214(11):3151–69. doi: 10.1084/jem.20171406 ; PubMed Central PMCID: PMCPMC5679168. 29061693 10.1084/jem.20171406 PMC5679168

[5] Salvalaio M, Rigon L, Belletti D, D'Avanzo F, Pederzoli F, Ruozi B, et al Targeted Polymeric Nanoparticles for Brain Delivery of High Molecular Weight Molecules in Lysosomal Storage Disorders. PloS one. 2016;11(5):e0156452 doi: 10.1371/journal.pone.0156452 ; PubMed Central PMCID: PMCPMC4881964. 27228099 10.1371/journal.pone.0156452 PMC4881964

[6] de Wit NM, Vanmol J, Kamermans A, Hendriks J, de Vries HE. Inflammation at the blood-brain barrier: The role of liver X receptors. Neurobiology of disease. 2017;107:57–65. doi: 10.1016/j.nbd.2016.09.015 . 27659108 10.1016/j.nbd.2016.09.015

[7] Banks WA. Blood-brain barrier as a regulatory interface. Forum of nutrition. 2010;63:102–10. Epub 2009/12/04. doi: 10.1159/000264398 . 19955778 10.1159/000264398

[8] Jones AR, Shusta EV. Blood-brain barrier transport of therapeutics via receptor-mediation. Pharmaceutical research. 2007;24(9):1759–71. doi: 10.1007/s11095-007-9379-0 ; PubMed Central PMCID: PMC2685177. 17619996 10.1007/s11095-007-9379-0 PMC2685177

[9] Toth A, Veszelka S, Nakagawa S, Niwa M, Deli MA. Patented in vitro blood-brain barrier models in CNS drug discovery. Recent Pat CNS Drug Discov. 2011;6(2):107–18. Epub 2011/05/19. . 21585327 10.2174/157488911795933910

[10] Kaisar MA, Sajja RK, Prasad S, Abhyankar VV, Liles T, Cucullo L. New experimental models of the blood-brain barrier for CNS drug discovery. Expert Opin Drug Discov. 2017;12(1):89–103. Epub 2016/10/27. doi: 10.1080/17460441.2017.1253676 ; PubMed Central PMCID: PMCPMC5521006. 27782770 10.1080/17460441.2017.1253676 PMC5521006

[11] Helms HC, Abbott NJ, Burek M, Cecchelli R, Couraud PO, Deli MA, et al In vitro models of the blood-brain barrier: An overview of commonly used brain endothelial cell culture models and guidelines for their use. Journal of cerebral blood flow and metabolism: official journal of the International Society of Cerebral Blood Flow and Metabolism. 2016;36(5):862–90. doi: 10.1177/0271678X16630991 ; PubMed Central PMCID: PMC4853841. 26868179 10.1177/0271678X16630991 PMC4853841

[12] Yousif S, Marie-Claire C, Roux F, Scherrmann JM, Decleves X. Expression of drug transporters at the blood-brain barrier using an optimized isolated rat brain microvessel strategy. Brain research. 2007;1134(1):1–11. doi: 10.1016/j.brainres.2006.11.089 . 17196184 10.1016/j.brainres.2006.11.089

[13] Bowman PD, Betz AL, Ar D, Wolinsky JS, Penney JB, Shivers RR, et al Primary culture of capillary endothelium from rat brain. In vitro. 1981;17(4):353–62. . 6263791 10.1007/BF02618147

[14] Emmert-Buck MR, Bonner RF, Smith PD, Chuaqui RF, Zhuang Z, Goldstein SR, et al Laser capture microdissection. Science. 1996;274(5289):998–1001. . 8875945 10.1126/science.274.5289.998

[15] Gomez-Zepeda D, Chaves C, Taghi M, Sergent P, Liu WQ, Chhuon C, et al Targeted unlabeled multiple reaction monitoring analysis of cell markers for the study of sample heterogeneity in isolated rat brain cortical microvessels. J Neurochem. 2017;142(4):597–609. doi: 10.1111/jnc.14095 . 28581635 10.1111/jnc.14095

[16] Porte B, Chatelain C, Hardouin J, Derambure C, Zerdoumi Y, Hauchecorne M, et al Proteomic and transcriptomic study of brain microvessels in neonatal and adult mice. PloS one. 2017;12(1):e0171048 Epub 2017/02/01. doi: 10.1371/journal.pone.0171048 ; PubMed Central PMCID: PMCPMC5283732. 28141873 10.1371/journal.pone.0171048 PMC5283732

[17] Peirson SN, Butler JN, Foster RG. Experimental validation of novel and conventional approaches to quantitative real-time PCR data analysis. Nucleic acids research. 2003;31(14):e73 ; PubMed Central PMCID: PMC167648. 12853650 10.1093/nar/gng073 PMC167648

[18] Zhai Z, Yao Y, Wang Y. Importance of suitable reference gene selection for quantitative RT-PCR during ATDC5 cells chondrocyte differentiation. PloS one. 2013;8(5):e64786 doi: 10.1371/journal.pone.0064786 ; PubMed Central PMCID: PMC3660368. 23705012 10.1371/journal.pone.0064786 PMC3660368

[19] Chechi K, Gelinas Y, Mathieu P, Deshaies Y, Richard D. Validation of reference genes for the relative quantification of gene expression in human epicardial adipose tissue. PloS one. 2012;7(4):e32265 doi: 10.1371/journal.pone.0032265 ; PubMed Central PMCID: PMC3325221. 22511915 10.1371/journal.pone.0032265 PMC3325221

[20] Kong Q, Yuan J, Gao L, Zhao L, Cheng F, Huang Y, et al Evaluation of Appropriate Reference Genes for Gene Expression Normalization during Watermelon Fruit Development. PloS one. 2015;10(6):e0130865 doi: 10.1371/journal.pone.0130865 ; PubMed Central PMCID: PMC4481515. 26110539 10.1371/journal.pone.0130865 PMC4481515

[21] Rydbirk R, Folke J, Winge K, Aznar S, Pakkenberg B, Brudek T. Assessment of brain reference genes for RT-qPCR studies in neurodegenerative diseases. Scientific reports. 2016;6:37116 doi: 10.1038/srep37116 ; PubMed Central PMCID: PMC5112547. 27853238 10.1038/srep37116 PMC5112547

[22] Chapman JR, Waldenstrom J. With Reference to Reference Genes: A Systematic Review of Endogenous Controls in Gene Expression Studies. PloS one. 2015;10(11):e0141853 Epub 2015/11/12. doi: 10.1371/journal.pone.0141853 ; PubMed Central PMCID: PMCPMC4640531. 26555275 10.1371/journal.pone.0141853 PMC4640531

[23] De Spiegelaere W, Dern-Wieloch J, Weigel R, Schumacher V, Schorle H, Nettersheim D, et al Reference gene validation for RT-qPCR, a note on different available software packages. PloS one. 2015;10(3):e0122515 doi: 10.1371/journal.pone.0122515 ; PubMed Central PMCID: PMC4380439. 25825906 10.1371/journal.pone.0122515 PMC4380439

[24] Taki FA, Abdel-Rahman AA, Zhang B. A comprehensive approach to identify reliable reference gene candidates to investigate the link between alcoholism and endocrinology in Sprague-Dawley rats. PloS one. 2014;9(5):e94311 doi: 10.1371/journal.pone.0094311 ; PubMed Central PMCID: PMC4019588. 24824616 10.1371/journal.pone.0094311 PMC4019588

[25] Vandesompele J, De Preter K, Pattyn F, Poppe B, Van Roy N, De Paepe A, et al Accurate normalization of real-time quantitative RT-PCR data by geometric averaging of multiple internal control genes. Genome biology. 2002;3(7):RESEARCH0034. ; PubMed Central PMCID: PMC126239. 12184808 10.1186/gb-2002-3-7-research0034 PMC126239

[26] Pfaffl MW, Tichopad A, Prgomet C, Neuvians TP. Determination of stable housekeeping genes, differentially regulated target genes and sample integrity: BestKeeper—Excel-based tool using pair-wise correlations. Biotechnology letters. 2004;26(6):509–15. . 15127793 10.1023/b:bile.0000019559.84305.47

[27] Macdonald JA, Murugesan N, Pachter JS. Endothelial cell heterogeneity of blood-brain barrier gene expression along the cerebral microvasculature. Journal of neuroscience research. 2010;88(7):1457–74. Epub 2009/12/22. doi: 10.1002/jnr.22316 . 20025060 10.1002/jnr.22316

[28] Ohtsuki S, Yamaguchi H, Katsukura Y, Asashima T, Terasaki T. mRNA expression levels of tight junction protein genes in mouse brain capillary endothelial cells highly purified by magnetic cell sorting. J Neurochem. 2008;104(1):147–54. doi: 10.1111/j.1471-4159.2007.05008.x . 17971126 10.1111/j.1471-4159.2007.05008.x

[29] Zlokovic BV. The blood-brain barrier in health and chronic neurodegenerative disorders. Neuron. 2008;57(2):178–201. doi: 10.1016/j.neuron.2008.01.003 . 18215617 10.1016/j.neuron.2008.01.003

[30] Dai M, Han J, El-Amouri SS, Brady RO, Pan D. Platelets are efficient and protective depots for storage, distribution, and delivery of lysosomal enzyme in mice with Hurler syndrome. Proc Natl Acad Sci U S A. 2014;111(7):2680–5. doi: 10.1073/pnas.1323155111 ; PubMed Central PMCID: PMCPMC3932929. 24550296 10.1073/pnas.1323155111 PMC3932929

[31] Wang D, Zhang W, Kalfa TA, Grabowski G, Davies S, Malik P, et al Reprogramming erythroid cells for lysosomal enzyme production leads to visceral and CNS cross-correction in mice with Hurler syndrome. Proc Natl Acad Sci U S A. 2009;106(47):19958–63. doi: 10.1073/pnas.0908528106 ; PubMed Central PMCID: PMCPMC2785274. 19903883 10.1073/pnas.0908528106 PMC2785274

[32] Dogrukol-Ak D, Kumar VB, Ryerse JS, Farr SA, Verma S, Nonaka N, et al Isolation of peptide transport system-6 from brain endothelial cells: therapeutic effects with antisense inhibition in Alzheimer and stroke models. Journal of cerebral blood flow and metabolism: official journal of the International Society of Cerebral Blood Flow and Metabolism. 2009;29(2):411–22. Epub 2008/11/13. doi: 10.1038/jcbfm.2008.131 . 19002200 10.1038/jcbfm.2008.131

[33] El-Amouri SS, Cao P, Miao C, Pan D. Secreted luciferase for in vivo evaluation of systemic protein delivery in mice. Mol Biotechnol. 2013;53(1):63–73. doi: 10.1007/s12033-012-9519-6 ; PubMed Central PMCID: PMCPMC4040271. 22407720 10.1007/s12033-012-9519-6 PMC4040271

[34] Clarke LA, Russell CS, Pownall S, Warrington CL, Borowski A, Dimmick JE, et al Murine mucopolysaccharidosis type I: targeted disruption of the murine alpha-L-iduronidase gene. Human molecular genetics. 1997;6(4):503–11. Epub 1997/04/01. . 9097952 10.1093/hmg/6.4.503

[35] Pan D, Sciascia A 2nd, Vorhees CV, Williams MT. Progression of multiple behavioral deficits with various ages of onset in a murine model of Hurler syndrome. Brain research. 2008;1188:241–53. doi: 10.1016/j.brainres.2007.10.036 ; PubMed Central PMCID: PMCPMC2323205. 18022143 10.1016/j.brainres.2007.10.036 PMC2323205

[36] Robertson RT, Levine ST, Haynes SM, Gutierrez P, Baratta JL, Tan Z, et al Use of labeled tomato lectin for imaging vasculature structures. Histochemistry and cell biology. 2015;143(2):225–34. doi: 10.1007/s00418-014-1301-3 . 25534591 10.1007/s00418-014-1301-3

[37] Bustin SA, Benes V, Garson J, Hellemans J, Huggett J, Kubista M, et al The need for transparency and good practices in the qPCR literature. Nature methods. 2013;10(11):1063–7. doi: 10.1038/nmeth.2697 . 24173381 10.1038/nmeth.2697

[38] Everaert BR, Boulet GA, Timmermans JP, Vrints CJ. Importance of suitable reference gene selection for quantitative real-time PCR: special reference to mouse myocardial infarction studies. PloS one. 2011;6(8):e23793 doi: 10.1371/journal.pone.0023793 ; PubMed Central PMCID: PMC3157472. 21858224 10.1371/journal.pone.0023793 PMC3157472

[39] Pinto F, Pacheco CC, Ferreira D, Moradas-Ferreira P, Tamagnini P. Selection of suitable reference genes for RT-qPCR analyses in cyanobacteria. PloS one. 2012;7(4):e34983 doi: 10.1371/journal.pone.0034983 ; PubMed Central PMCID: PMC3319621. 22496882 10.1371/journal.pone.0034983 PMC3319621

[40] Wen S, Chen X, Xu F, Sun H. Validation of Reference Genes for Real-Time Quantitative PCR (qPCR) Analysis of Avibacterium paragallinarum. PloS one. 2016;11(12):e0167736 doi: 10.1371/journal.pone.0167736 ; PubMed Central PMCID: PMC5152862. 27942007 10.1371/journal.pone.0167736 PMC5152862

[41] Takamori LM, Pereira AVC, Maia Souza G, Vieira LGE, Ferreira Ribas A. Identification of Endogenous Reference Genes for RT-qPCR Expression Analysis in Urochloa brizantha Under Abiotic Stresses. Scientific reports. 2017;7(1):8502 doi: 10.1038/s41598-017-09156-7 ; PubMed Central PMCID: PMC5561021. 28819216 10.1038/s41598-017-09156-7 PMC5561021

[42] Wang C, Cui HM, Huang TH, Liu TK, Hou XL, Li Y. Identification and Validation of Reference Genes for RT-qPCR Analysis in Non-Heading Chinese Cabbage Flowers. Frontiers in plant science. 2016;7:811 doi: 10.3389/fpls.2016.00811 ; PubMed Central PMCID: PMC4901065. 27375663 10.3389/fpls.2016.00811 PMC4901065

[43] Martins PK, Mafra V, de Souza WR, Ribeiro AP, Vinecky F, Basso MF, et al Selection of reliable reference genes for RT-qPCR analysis during developmental stages and abiotic stress in Setaria viridis. Scientific reports. 2016;6:28348 doi: 10.1038/srep28348 ; PubMed Central PMCID: PMC4913262. 27321675 10.1038/srep28348 PMC4913262

[44] Zhou Z, Cong P, Tian Y, Zhu Y. Using RNA-seq data to select reference genes for normalizing gene expression in apple roots. PloS one. 2017;12(9):e0185288 doi: 10.1371/journal.pone.0185288 . 28934340 10.1371/journal.pone.0185288 PMC5608369

[45] Drozd E, Krzyszton-Russjan J, Gruber B. Doxorubicin Treatment of Cancer Cells Impairs Reverse Transcription and Affects the Interpretation of RT-qPCR Results. Cancer genomics & proteomics. 2016;13(2):161–70. . 26912806

[46] Martin JL. Validation of Reference Genes for Oral Cancer Detection Panels in a Prospective Blinded Cohort. PloS one. 2016;11(7):e0158462 doi: 10.1371/journal.pone.0158462 ; PubMed Central PMCID: PMC4943624. 27411053 10.1371/journal.pone.0158462 PMC4943624

[47] Durrenberger PF, Fernando FS, Magliozzi R, Kashefi SN, Bonnert TP, Ferrer I, et al Selection of novel reference genes for use in the human central nervous system: a BrainNet Europe Study. Acta neuropathologica. 2012;124(6):893–903. doi: 10.1007/s00401-012-1027-z . 22864814 10.1007/s00401-012-1027-z

[48] Cheung TT, Weston MK, Wilson MJ. Selection and evaluation of reference genes for analysis of mouse (Mus musculus) sex-dimorphic brain development. PeerJ. 2017;5:e2909 doi: 10.7717/peerj.2909 ; PubMed Central PMCID: PMC5251938. 28133578 10.7717/peerj.2909 PMC5251938

[49] Pfaffl MW. A new mathematical model for relative quantification in real-time RT-PCR. Nucleic acids research. 2001;29(9):e45 ; PubMed Central PMCID: PMCPMC55695. 11328886 10.1093/nar/29.9.e45 PMC55695

[50] Andersen CL, Jensen JL, Orntoft TF. Normalization of real-time quantitative reverse transcription-PCR data: a model-based variance estimation approach to identify genes suited for normalization, applied to bladder and colon cancer data sets. Cancer research. 2004;64(15):5245–50. doi: 10.1158/0008-5472.CAN-04-0496 . 15289330 10.1158/0008-5472.CAN-04-0496

[51] Hellemans J, Mortier G, De Paepe A, Speleman F, Vandesompele J. qBase relative quantification framework and software for management and automated analysis of real-time quantitative PCR data. Genome biology. 2007;8(2):R19 doi: 10.1186/gb-2007-8-2-r19 ; PubMed Central PMCID: PMC1852402. 17291332 10.1186/gb-2007-8-2-r19 PMC1852402

[52] Spence EF, Soderling SH. Actin Out: Regulation of the Synaptic Cytoskeleton. The Journal of biological chemistry. 2015;290(48):28613–22. doi: 10.1074/jbc.R115.655118 ; PubMed Central PMCID: PMC4661376. 26453304 10.1074/jbc.R115.655118 PMC4661376

[53] Schroeder A, Mueller O, Stocker S, Salowsky R, Leiber M, Gassmann M, et al The RIN: an RNA integrity number for assigning integrity values to RNA measurements. BMC molecular biology. 2006;7:3 doi: 10.1186/1471-2199-7-3 ; PubMed Central PMCID: PMC1413964. 16448564 10.1186/1471-2199-7-3 PMC1413964

[54] Fleige S, Walf V, Huch S, Prgomet C, Sehm J, Pfaffl MW. Comparison of relative mRNA quantification models and the impact of RNA integrity in quantitative real-time RT-PCR. Biotechnology letters. 2006;28(19):1601–13. doi: 10.1007/s10529-006-9127-2 . 16900335 10.1007/s10529-006-9127-2

[55] Pan D, Kalfa TA, Wang D, Risinger M, Crable S, Ottlinger A, et al K-Cl cotransporter gene expression during human and murine erythroid differentiation. The Journal of biological chemistry. 2011;286(35):30492–503. doi: 10.1074/jbc.M110.206516 ; PubMed Central PMCID: PMC3162409. 21733850 10.1074/jbc.M110.206516 PMC3162409

[56] Duda J, Fauler M, Grundemann J, Liss B. Cell-Specific RNA Quantification in Human SN DA Neurons from Heterogeneous Post-mortem Midbrain Samples by UV-Laser Microdissection and RT-qPCR. Methods Mol Biol. 2018;1723:335–60. Epub 2018/01/19. doi: 10.1007/978-1-4939-7558-7_19 . 29344870 10.1007/978-1-4939-7558-7_19

[57] Buh Gasparic M, Tengs T, La Paz JL, Holst-Jensen A, Pla M, Esteve T, et al Comparison of nine different real-time PCR chemistries for qualitative and quantitative applications in GMO detection. Analytical and bioanalytical chemistry. 2010;396(6):2023–9. doi: 10.1007/s00216-009-3418-0 . 20087729 10.1007/s00216-009-3418-0

[58] Navarro E, Serrano-Heras G, Castano MJ, Solera J. Real-time PCR detection chemistry. Clinica chimica acta; international journal of clinical chemistry. 2015;439:231–50. doi: 10.1016/j.cca.2014.10.017 . 25451956 10.1016/j.cca.2014.10.017

[59] Sanjuan-Jimenez R, Colmenero JD, Morata P. Lessons learned with molecular methods targeting the BCSP-31 membrane protein for diagnosis of human brucellosis. Clinica chimica acta; international journal of clinical chemistry. 2017;469:1–9. doi: 10.1016/j.cca.2017.03.014 . 28315659 10.1016/j.cca.2017.03.014

[60] Paul D, Cowan AE, Ge S, Pachter JS. Novel 3D analysis of Claudin-5 reveals significant endothelial heterogeneity among CNS microvessels. Microvascular research. 2013;86:1–10. doi: 10.1016/j.mvr.2012.12.001 ; PubMed Central PMCID: PMC3570614. 23261753 10.1016/j.mvr.2012.12.001 PMC3570614

[61] Zlokovic BV. Neurovascular pathways to neurodegeneration in Alzheimer's disease and other disorders. Nature reviews Neuroscience. 2011;12(12):723–38. doi: 10.1038/nrn3114 ; PubMed Central PMCID: PMC4036520. 22048062 10.1038/nrn3114 PMC4036520

[62] Menezes MJ, McClenahan FK, Leiton CV, Aranmolate A, Shan X, Colognato H. The extracellular matrix protein laminin alpha2 regulates the maturation and function of the blood-brain barrier. The Journal of neuroscience: the official journal of the Society for Neuroscience. 2014;34(46):15260–80. doi: 10.1523/JNEUROSCI.3678-13.2014 . 25392494 10.1523/JNEUROSCI.3678-13.2014 PMC6608454

[63] Ioannidou S, Deinhardt K, Miotla J, Bradley J, Cheung E, Samuelsson S, et al An in vitro assay reveals a role for the diaphragm protein PV-1 in endothelial fenestra morphogenesis. Proc Natl Acad Sci U S A. 2006;103(45):16770–5. doi: 10.1073/pnas.0603501103 ; PubMed Central PMCID: PMC1636530. 17075074 10.1073/pnas.0603501103 PMC1636530

[64] Daneman R, Zhou L, Kebede AA, Barres BA. Pericytes are required for blood-brain barrier integrity during embryogenesis. Nature. 2010;468(7323):562–6. doi: 10.1038/nature09513 ; PubMed Central PMCID: PMC3241506. 20944625 10.1038/nature09513 PMC3241506

[65] Shue EH, Carson-Walter EB, Liu Y, Winans BN, Ali ZS, Chen J, et al Plasmalemmal vesicle associated protein-1 (PV-1) is a marker of blood-brain barrier disruption in rodent models. BMC neuroscience. 2008;9:29 doi: 10.1186/1471-2202-9-29 ; PubMed Central PMCID: PMC2270280. 18302779 10.1186/1471-2202-9-29 PMC2270280

[66] Yu D, Corbett B, Yan Y, Zhang GX, Reinhart P, Cho SJ, et al Early cerebrovascular inflammation in a transgenic mouse model of Alzheimer's disease. Neurobiology of aging. 2012;33(12):2942–7. doi: 10.1016/j.neurobiolaging.2012.02.023 . 22440674 10.1016/j.neurobiolaging.2012.02.023

[67] Sweeney MD, Ayyadurai S, Zlokovic BV. Pericytes of the neurovascular unit: key functions and signaling pathways. Nature neuroscience. 2016;19(6):771–83. doi: 10.1038/nn.4288 . 27227366 10.1038/nn.4288 PMC5745011

[68] Zyzynska-Granica B, Koziak K. Identification of suitable reference genes for real-time PCR analysis of statin-treated human umbilical vein endothelial cells. PloS one. 2012;7(12):e51547 doi: 10.1371/journal.pone.0051547 ; PubMed Central PMCID: PMC3519707. 23251572 10.1371/journal.pone.0051547 PMC3519707

