# Supplementary material for: Comprehensive evaluation of blood-brain barrier-forming micro-vasculatures: Reference and marker genes with cellular composition
Source: PLoS One. 2018 May 15;13(5):e0197379. doi: 10.1371/journal.pone.0197379 (PMC5953434; doi:10.1371/journal.pone.0197379)
Supplement: S1 Fig — (PPTX) [file pone.0197379.s001.pptx]

## Slide 1
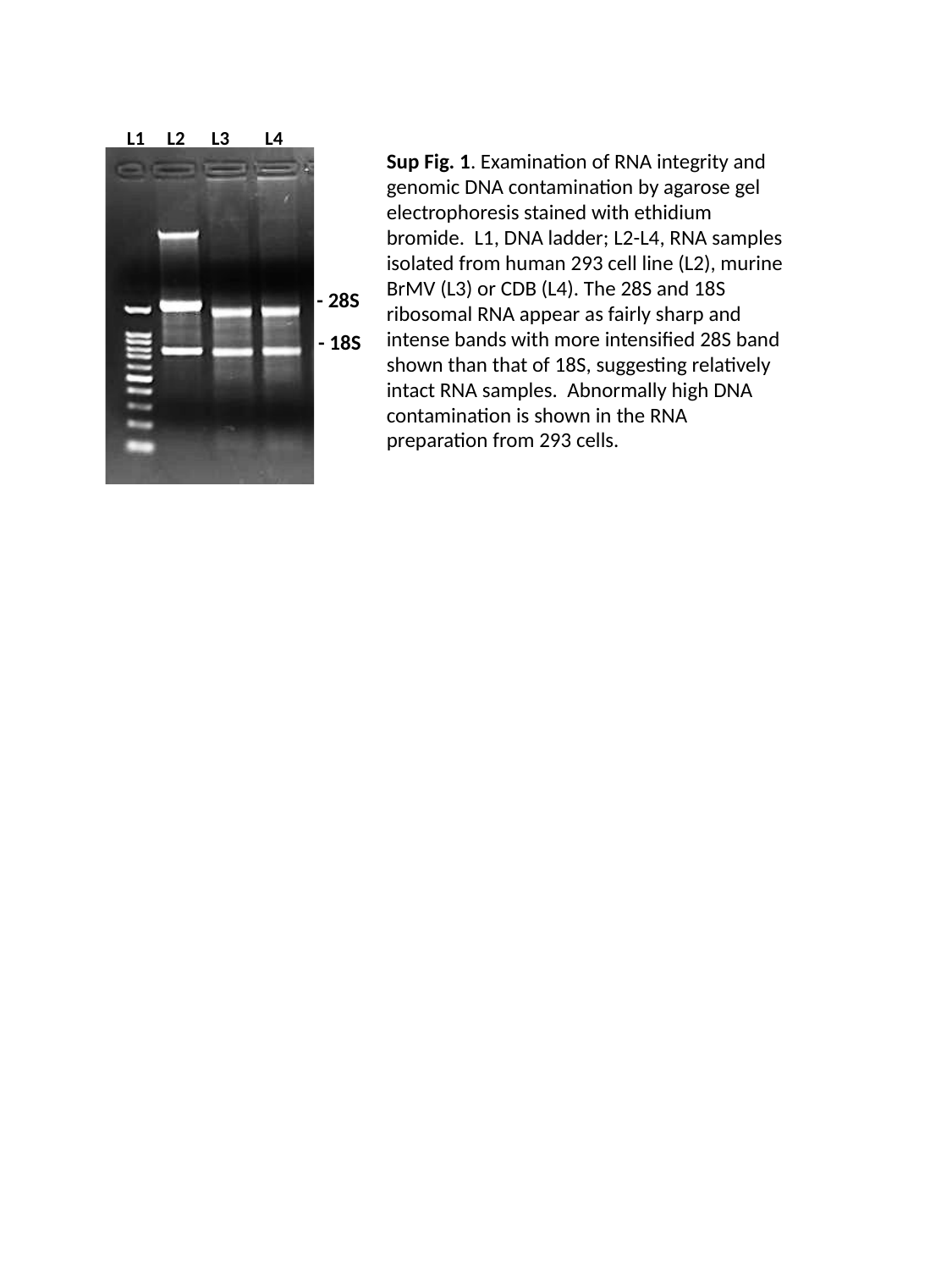

L1 L2 L3 L4
- 28S
- 18S
Sup Fig. 1. Examination of RNA integrity and genomic DNA contamination by agarose gel electrophoresis stained with ethidium bromide. L1, DNA ladder; L2-L4, RNA samples isolated from human 293 cell line (L2), murine BrMV (L3) or CDB (L4). The 28S and 18S ribosomal RNA appear as fairly sharp and intense bands with more intensified 28S band shown than that of 18S, suggesting relatively intact RNA samples. Abnormally high DNA contamination is shown in the RNA preparation from 293 cells.
