# Supplementary material for: Comprehensive evaluation of blood-brain barrier-forming micro-vasculatures: Reference and marker genes with cellular composition
Source: PLoS One. 2018 May 15;13(5):e0197379. doi: 10.1371/journal.pone.0197379 (PMC5953434; doi:10.1371/journal.pone.0197379)
Supplement: S1 Table — (DOCX) [file pone.0197379.s002.docx]

**Supplemental Table 1. Evaluation of repeatability and accuracy of each real-time qPCR setting for reference genes using 3T3 cells**. The deltaRn was set at 0.02 for all reference genes, except for *Gapdh* at 0.03 due to higher background noise.
